# An endophytic Basidiomycete, *Grammothele lineata*, isolated from *Corchorus olitorius*, produces paclitaxel that shows cytotoxicity

**DOI:** 10.1371/journal.pone.0178612

**Published:** 2017-06-21

**Authors:** Avizit Das, Mohammad Imtiazur Rahman, Ahlan Sabah Ferdous, Al- Amin, Mohammad Mahbubur Rahman, Nilufar Nahar, Md. Aftab Uddin, Mohammad Riazul Islam, Haseena Khan

**Affiliations:** 1 Department of Biochemistry and Molecular Biology, University of Dhaka, Dhaka, Bangladesh; 2 Development of Fiber and Polymer Science Laboratory, BCSIR, Dhaka, Bangladesh; 3 Department of Chemistry, University of Dhaka, Dhaka, Bangladesh; 4 Department of Genetic Engineering and Biotechnology, University of Dhaka, Dhaka, Bangladesh; Tallinn University of Technology, ESTONIA

## Abstract

*Grammothele lineata*, an endophyte isolated in our laboratory from jute (*Corchorus olitorius* acc. 2015) was found to be a substantial paclitaxel producer. Taxol and its related compounds, produced by this endophyte were extracted by growing the fungus in simple nutrient media (potato dextrose broth, PDB). Taxol was identified and characterized by different analytical techniques (TLC, HPLC, FTIR, LC-ESI-MS/MS) following its extraction by ethyl acetate. In PDB media, this fungus was found to produce 382.2 μgL^-1^ of taxol which is about 7.6 x10^3^ fold higher than the first reported endophytic fungi, *Taxomyces andreanae*. The extracted taxol exhibited cytotoxic activity in an *in vitro* culture of HeLa cancer cell line. The fungal extract also exhibited antifungal and antibacterial activities against different pathogenic strains. This is the first report of a jute endophytic fungus harboring the capacity to produce taxol and also the first reported taxol producing species that belongs to the Basidiomycota phylum, so far unknown to be a taxol producer. These findings suggest that the fungal endophyte, *Grammothele lineata* can be an excellent source of taxol and can also serve as a potential species for chemical and genetic engineering to enhance further the production of taxol.

## Introduction

It is generally assumed that fungal endophytes have the capacity to produce bioactive compounds and can independently synthesize secondary metabolites similar to those made by the host plants [1]. Endophytic fungi gained enormous attention when detection of taxol in the endophytic fungus (*Taxomyces andreanae*) isolated from yew plant (*Taxus brevifolia*) was first reported [2]. Paclitaxel (Taxol^®^), the first taxane isolated from natural sources (plant and fungi) has proven to be effective against a broad range of cancers, generally considered to be recalcitrant to conventional chemotherapy and is ranked the world’s first billion dollar anticancer drug [3]. However, production of taxol is still a challenge in medical and pharmaceutical sectors. 20 kg of the bark of tropical and temperate yews (*Taxus* species), a leading source of taxol is required for every mg of taxol [4]. It may be mentioned that 2.5–3.0 g of taxol is required for a full regimen in cancer treatment [5], which makes it quite expensive and unreachable for most people. At the same time, collection of compounds like taxol from a plant source is a slow process and would lead to destruction of their ecological habitat rendering conservation of such plants a necessity [6]. As such, there is a growing interest in alternative sources of taxol. Several methods have been developed for taxol production, namely total chemical synthesis [7, 8], semi-synthesis from its precursor [9] and plant tissue cell culture based production [10]. But the large number of reaction steps required for chemical synthesis is difficult to pursue and extraction of precursors for semi-synthesis of taxol is costly. At the same time, long incubation period, less biomass, low yield and genetic instability of plant tissue cell culture based methods make all the three procedures inefficient. Since the description of the first taxol producing fungus [2], microorganisms are being explored as potential replacements for an environmentally acceptable, comparatively simple and inexpensive method of taxol production [6]. Although the amount of taxol produced by endophytic fungi is relatively small in comparison to that of plants, fast growth at high cell density cultivation and the possibility of scale-up on an industrial level make endophytic fungi a promising alternative [11]. Microorganisms which are said to produce taxol are primarily but not exclusively- endophytes of plants known to harbor some form of medicinal value [12]. Some of the taxol producing fungi are soil borne [13] or even plant pathogens [14]. Moreover, few bacteria have also been mentioned to yield taxol [11]. Many publications and their resulting patents have been reported regarding the biosynthesis of taxol and related taxanes by microorganisms [15], of which a recent one is a taxol producing fungus from dermatitic scurf of the Giant Panda [16].

Jute (*Corchorus* sp.), an annual dicotyledonous crop is known mostly for its high quality tensile natural fiber [17]. Although not reported to produce taxol, *C*. *olitorius* has long been recognized as a medicinal herb and its extract is known to have apoptotic activity on tumor cell lines [18]. In addition, *C*. *olitorius* has also been described to possess promising antibacterial and antifungal activity [19]. A recent study has found a diverse community of endophytic fungi in *C*. *olitorius* [20]. Further unrevealing of jute endophytes and gaining an understanding of the antitumor activity of jute extracts framed the background of our current work. The objective of this study was to identify by molecular, analytical, spectral and bio-assay based methods, jute endophytic fungi having an independent capacity to produce taxol. Three jute endophytic fungal isolates were initially found to be positive for the genes of taxol biosynthesis pathway. One among the three is SDL-CO-2015-1, a Basidiomycete capable of producing taxol ascertained by TLC, HPLC, LC-ESI-MS/MS and FTIR. The isolated taxol was effective against HeLa cancer cell line and the fungal extract was found to be promising in antimicrobial screening as well. This endofungus SDL-CO-2015-1, identified as *Grammothele lineata* is the first ever Basidiomycete found to possess a capacity for taxol production.

## Materials and methods

### Collection of plant samples and isolation of endophytic fungi

Fresh plant samples (root, stem, leaf, flower and seed) of jute (*C*. *olitorius*) collected from the botanical garden of the University of Dhaka, were surface sterilized by washing under running tap water, rinsing with 70% ethanol for one min, then treating with 4% sodium hypochlorite for three min. Finally samples were soaked in autoclaved milli-Q water and dried on sterile filter paper [21]. The sterilized samples were then cut into small pieces using a sterile blade and incubated on a potato dextrose agar (PDA) (HIMEDIA^®^) plate (Petri plate, Scientific Systems, India) at 28°C. After 7–10 days of incubation, a mixed fungal culture appeared on the plate, from which pure and single culture plates for 25 individual fungi (data not shown) were generated through a repeated sub-culture. All processes were carried out under sterile conditions.

### Fungal DNA isolation

DNA from different fungal species was isolated using the modified SDS method [22]. In brief, fungal mycelia were crushed with liquid nitrogen followed by addition of lysis buffer containing 3% SDS, 50 mM Tris-Cl (pH 8.0), 50 mM EDTA and 2% mercaptoethanol. The thawed suspension was incubated for 60 min at 65°C and then centrifuged for 5 min at 5000 x g (Eppendorf Centrifuge 5810 R) to precipitate the cell debris. Supernatant was then mixed with an equal volume of phenol: chloroform: isoamylalcohol (25:24:1) mixture and centrifuged again for 10 min at 18,500 x g. Collection of supernatant was followed by addition of equal volume of chloroform: isoamylalcohol (24:1) mixture and centrifuged for 10 min at 18,500 x g. The final supernatant was then collected, 25 μL of 3 M Na-acetate was added, the volume made 1 mL with isopropanol and kept overnight at -30°C. The suspension was centrifuged the next day for 10 min at 18,500 x g to collect the DNA pellet. This pellet was dissolved in 300 μL TE buffer and kept at 65°C for 15 min. Next 15 μL of 2 M Na-acetate and 100% ethanol was added to make the volume 1mL. Centrifugation was again carried out at 18,500 x g for 10 min. Supernatant was discarded and the pellet was washed with 70% ethanol. The pellet was finally dried and dissolved in TE buffer. Concentration and purity of the DNAs were checked using a Nanodrop (ND-1000).

### PCR based molecular screening for taxol producing endophytic fungi

Primary search for taxol producing fungi was PCR based, using specific primers for three key genes of the taxol biosynthetic pathway in a GeneAmpR PCR System 9700 (Applied Biosystem). The genes screened were- *ts* encoding a rate limiting enzyme taxadiene synthase (*ts*-F: ATCAGTCCGTCTGCATACGACA, *ts*-R: TAAGCCTGGCTTCCC GTGTTGT), *dbat* encoding a 10-deacetylbaccatin III-10-O-acetyl transferase (*dbat*-F: ATGGCTGAC ACTGACCTCTCAGT, *dbat*-R: GGCCTGCTCCTAGTCCATCACAT) and *bapt* encoding a C-13 phenyl propanoid side chain-CoA acyltransferase or *bapt* (*bapt*-F: CCTCTCTCCGCCATTGACAA CAT, bapt-R: GTCGCTGTCAGCCATGGCTT) [23, 24]. For a reaction volume of 15 μL 50 ng of DNA sample was used together with, 0.33 μM of specific primers, 1X *Taq* buffer, 200 μM dNTPs and 0.375U *Taq* DNA polymerase. Positive samples *i*.*e*. samples with distinct amplicons for the different primer sets after electrophoresis in 1% agarose gel were then subjected to gel extraction using the PureLink^™^ Quick Gel Extraction Kit (Invitrogen, Germany) followed by single pass sequencing (1^st^ Base Laboratories, Malaysia). Sequence data were analyzed using BLAST (www.ncbi.nlm.nih.gov/BLAST).

### Identification and characterization of fungal isolate

One of the fungal isolates SDL-CO-2015-1 positive in genetic screening, was then subjected to macroscopic, microscopic and molecular identifications.

#### Morphological characterization of the fungus

For macroscopic observation, the fungus was grown on 90 mm disposable petri plates containing PDA. The fungal culture was monitored continuously from the day of inoculation until the plates were fully covered with mature mycelia and spores.

Microscopic study was carried out with fungal parts stained with lactophenol aniline blue (Sigma, Germany), 5% KOH solution and observed under an inverted fluorescent microscope (EVOS FL, ThermoFisher Scientific, USA).

#### Molecular identification of SDL-CO-2015-1

For molecular identification, ITS (internal transcribed spacer region) primers, ITS-1 (5′-TCCGTAGGTGAACCTGCG-3′) and ITS-4: (5′-TCCTCCGCTTATTGATATGC-3′) which amplify the highly variable ITS1 and ITS2 sequences surrounding the 5.8S-coding sequence and situated between the Small Sub-Unit-coding sequence (SSU) and the Large Sub-Unit-coding sequence (LSU) of the ribosomal operon were used [25]. PCR amplicons were analyzed in the same way as mentioned earlier. ITS sequence of the endophytic fungi was used to estimate phylogenetic relationship by the maximum likelihood method using the MEGA software (version 7.0, Biodesign Institute, USA). Bootstrap analyses were based on 1000 replicates to assess the level of confidence of each node in the gene tree [26].

### Preparation of fungal extracts and isolation and identification of taxol

Fungal isolates were inoculated in 500 mL Erlenmeyer flasks containing 300 mL of potato dextrose broth (PDB) (potato 200 g/L and glucose 20 g/L) and allowed to grow at 28°C in a shaking incubator (180 rpm). After 21 days, mycelia (intracellular component) and culture media (extracellular component) were separated by filtration. Mycelia was next homogenized and then both the extra and intracellular components were individually extracted twice with equal volume of ethyl acetate. The extracts were evaporated in a rotary evaporator at 35°C under reduced pressure at 250 psi. These crude extracts were then dissolved in methanol and used in chromatographic separation and spectroscopic analysis.

The extracts were subjected to thin layer chromatographic (TLC) analysis using a Macherey Nagel & Co. KG 0.2 mm (20×20 cm) silica gel pre-coated plate where ethyl acetate: 2-propanol (95:5, v/v) was used as the mobile phase. Samples were loaded at one end, 1 cm from the edge along with the standard (commercial paclitaxel, Sigma-Aldrich). Presence of fungal taxol was detected with 1% vanillin/sulphuric acid (w/v). The R_f_ values were then calculated for the desired bands.

Analytical HPLC Dionex Ultimate 3000 with a C18 column (Nucleodur, 250x4.6 mm, particle size 5μm, pore size 110Å, 100-5C18ec) was used to analyze the fungal extract along with the standard taxol. Standard taxol and lyophilized fungal extract were dissolved in HPLC grade methanol, filtered and 20 μL was injected to the column [23]. A multi-step gradient system was performed with (0–2 min, 20% methanol; 2–4 min, 30% methanol; 4–30 min, 30–80% methanol; 30–33 min, 80–100% methanol; 33–38 min, 100–20% methanol; volume fraction) an optimized protocol. Wavelength at 227 nm was used to detect compounds eluted from the column. The extract was at first compared with the standard taxol to assess if it contained taxol on the basis of its retention time in HPLC. The fraction with the same retention time as the standard was collected (mentioned in result), purified by semi-preparative HPLC and compared again with the standard taxol. Fungal extract was subjected to LC-ESI-MS/MS analysis to unravel the molecular mass of taxol. The LC separation was performed on Shimadzu Prominence Ultra Flow Liquid Chromatography (UFLC) using Shim-pack GISS C18 analytical column (4.6 X 250 mm, 5 μM) and an isocratic elution with methanol: water (60:40) for taxol at a flow rate of 1 mL min^-1^. Mass spectra were acquired by a Shimadzu Chromatograph Mass Spectrometer (LCMS-8050). An electron ionization device was used for sample analyses (nebulizing gas N_2,_ collision gas N_2,_ EI 70 eV). At first the methanolic fungal extract was analyzed through a scanning mode. A characteristic peak of taxol was found and its mass fragmentation patterns were analyzed along with standard taxol in LC-MRM-MS.

Infrared spectra were recorded on a FTIR spectrometer (Frontier PerkinElmer, UK) using purified sample. Interferograms were collected over the spectral range, 4000 cm^-1^ to 650 cm^-1^, with a nominal resolution of 2 cm^-1^ [27].

### Quantification of fungal taxol

Different concentrations of standard paclitaxel solutions were analyzed in HPLC in the same way as mentioned above for the construction of a standard curve. The peak areas of different concentrations of the standard solutions were used to quantify fungal taxol per liter of total culture.

### Cytotoxicity assay

HPLC purified fungal and standard taxol were first dissolved in 10% DMSO in Dulbecco’s modified Eagle’s medium (DMEM). HeLa cells were cultured in a 40 mL culture flask with DMEM supplemented with 1% penicillin-streptomycin (1:1), 0.2% gentamycin and 10% fetal bovine serum (FBS) and incubated at 37°C with 5% CO_2_ [28]. After 24 hr incubation cells were recovered from the culture flask by discarding the media followed by washing with PBS and incubated with 2 mL of 0.25% 1X trypsin-EDTA for 5 min at 37°C with 5% CO_2_. Next the cells were collected into a 50 mL falcon tube and centrifuged at 500 x g for 5 min. The resulting pellet was dissolved in 2 mL PBS and the cell number was counted in a hemocytometer using a trinocular microscope with a camera (Olympus, Japan). Cells (1x 10^4^ cells per well) in DMEM were seeded onto the wells of a 96-well plate and incubated 24 hr at 37°C with 5% CO_2_. Then 100 μL of purified taxol and standard taxol each at a concentration of 0.005 μM and 10% DMSO in DMEM (used as a vehicle control) were applied and incubated at 37°C with 5% CO_2_. After 24 hr the cells were collected, washed with PBS, stained with PI (propidium iodide) and analyzed with a fluorescence activated cell cytometer (FACs). Duplicates were used for both the sample and the standard.

### Antimicrobial assay

Antimicrobial activities of both extra- and intracellular extracts (prepared according to the method mentioned above) were assayed against indicator bacterial and fungal strains by well diffusion assay. In this study, two pathogenic fungal strains, *Macrophomina phaseolina* and *Aspergillus fumigatus* and two bacterial strains, gram positive *Staphylococcus aureus* and gram negative *Burkholderia sp* were used as indicator strains. 100 μL of the indicator bacterial and fungal suspensions were spread on tryptic soya agar (TSA) and PDA plates respectively. 6 mm diameter wells were then made in each plate with the help of a borer. 40 μL of different concentrations (Tables 1 and 2) of either intra or extracellular extracts of SDL-CO-2015-1 were loaded onto each well. The plates were then incubated for 24 hr at 37°C for antibacterial assay and 36 hr at 28°C for antifungal assay. The tests were done in duplicates.

**Table 1:** Measurement of antifungal activity for different amounts of intracellular extract of *G*. *lineata* in methanol by well diffusion method.

Sample	Amount of extract per well (μg)	Zone of inhibition in mm	
		*Aspergillus fumigatus*	*Macrophomina phaseolina*
**Intracellular**	Negative Control (no extract)	0±0.0^c^ (N/A)	0±0.0^e^ (N/A)
	200	18.5±0.71***^a^	24.5±0.71***^a^
	100	15.5±0.71***^b^	20.5±0.71***^b^
	50	0±0.0^c^ (N/A)	18.5±0.71***^c^
	25	0±0.0^c^ (N/A)	16±0.00***^d^
	12.5	0±0.0^c^(N/A)	14.5±0.71***^d^
	6.25	0±0.0^c^(N/A)	0±0.0^e^ (N/A)

*** denotes p value <0.001.

For each individual indicator organism, means that do not share a letter are significantly different.

**Table 2:** Measurement of antibacterial activity for different amounts of intracellular and extracellular extracts of *G*. *lineata* in methanol by well diffusion method.

Sample	Amount of extract per well (μg)	Zone of inhibition (mm)	
		*Staphylococcus aureus*	*Burkholderia sp*
**Intracellular extract**	Negative Control (no extract)	0 ±0.0^e^ (N/A)	0 ±0.0^f^ (N/A)
	200	26.5±0.71***^a^	37.5±0.71***^a^
	100	24±0.0***^b^	32.5±0.71***^b^
	50	22.5±0.71***^b^	28.0±1.41***^c^
	25	22±0.0***^b^	21.5±0.71***^d^
	12.5	20±0.0***^c^	15.0±1.41***^e^
	6.25	14.5±0.71***^d^	0 ±0.0^f^ (N/A)
	3.125	0 ±0.0^e^ (N/A)	0 ±0.0^f^ (N/A)
**Extracellular extract**	Negative Control (no extract)	0 ±0.0 (N/A)^e^	0 ±0.0^e^ (N/A)
	200	27±0.0***^a^	30.5±0.71***^a^
	100	22.5±0.71***^b^	27.0±0.0***^b^
	50	20±0.0***^c^	21.5±0.71***^c^
	25	15±0.0***^d^	16.5±0.71***^d^
	12.5	0 ±0.0^e^ (N/A)	0 ±0.0^e^ (N/A)
	6.25	0 ±0.0^e^ (N/A)	0 ±0.0^e^ (N/A)

*** Denotes p value <0.001.

For each individual indicator organism, means that do not share a letter are significantly different.

### Statistical analysis

Two independent replicates were taken into account for each test. Results are given as means ± standard deviation. Statistical analyses were done using one way ANOVA and Tukey’s test in R program (alpha value 0.05) with P value <0.001 considered as highly significant. ‘***’ denotes P<0.001. For each individual indicator organism, means that do not share a letter are significantly different.

## Results

**Fig 1:**
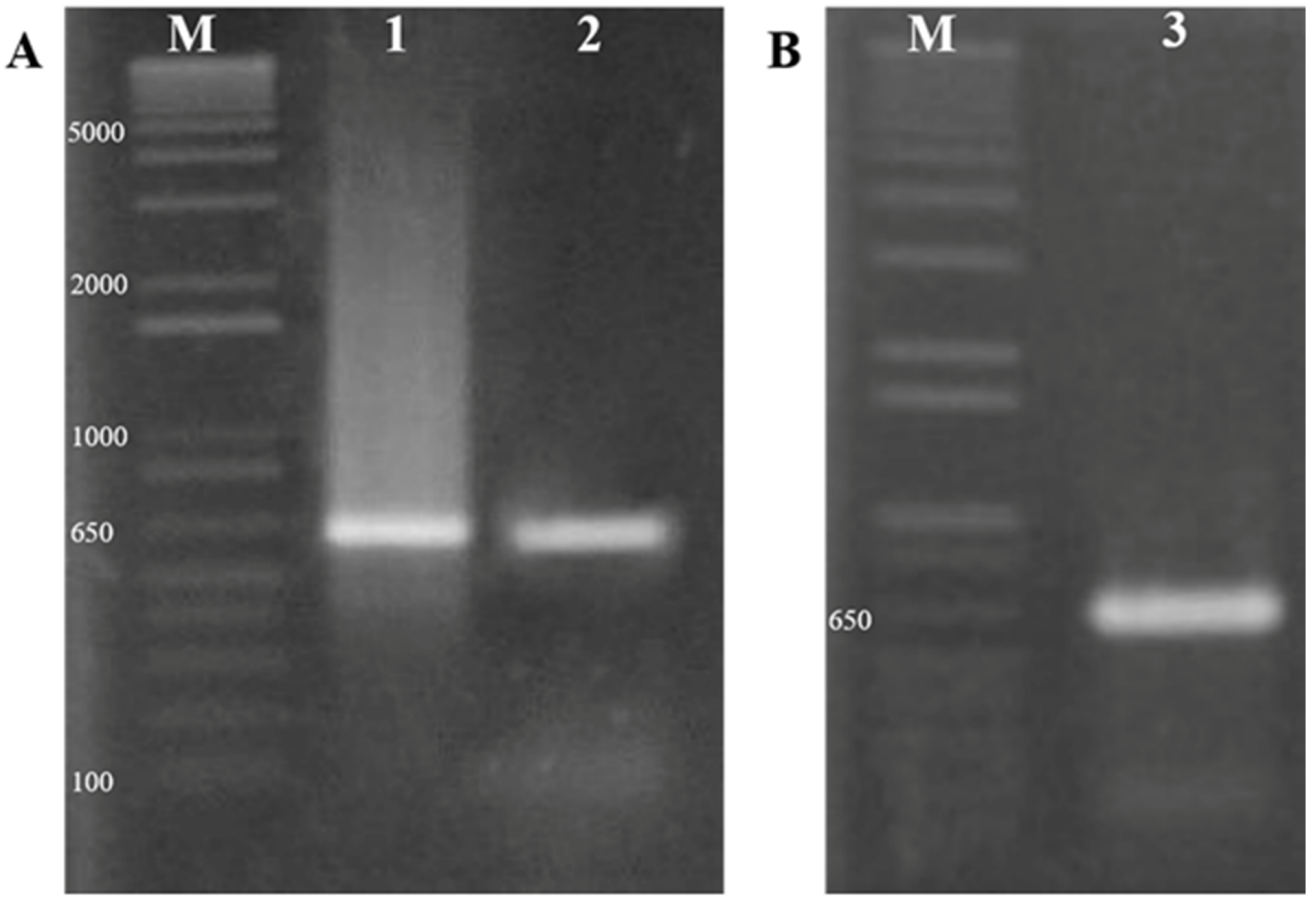
Screening for genes of taxol biosynthetic pathway and molecular identification of the fungal isolate. (A) SDL-CO-2015-1 shows a specific band of about 650bp approximately the expected size for both *ts* and *dbat* genes amplicons obtained specific primer pairs were used (M = 1kb+ Ladder, 1 = PCR amplicon for *ts* gene, 2 = PCR amplicon for *dbat* gene) in 1% agarose gel. (B) PCR amplicon for the ITS specific region used for molecular identification of the fungal isolate SDL-CO-2015-1 in 1% agarose gel (M = 1kb+ Ladder, 3 = PCR amplicon for ITS region) (for the sequence see Table A in S1 File).

A total of 25 fungal isolates were screened initially for the presence of taxol biosynthetic pathway genes, *ts*, *dbat* and *bapt*. DNA sequences of the genes available in the public database for fungi and plant were used in designing and synthsizing gene specific primers in order to find potential candidates. After PCR amplification, fungal isolate, SDL-CO-2015-1 was (with two others, data not shown) found to be PCR positive for *ts* and *dbat* gene (Fig 1A) (sequences provide Table A in S1 File). In our quest for a taxol producing endophyte it was decided to advance further with SDL-CO-2015-1.

### Morphological characterization of PCR positive fungal isolate, SDL-CO-2015-1

**Fig 2:**
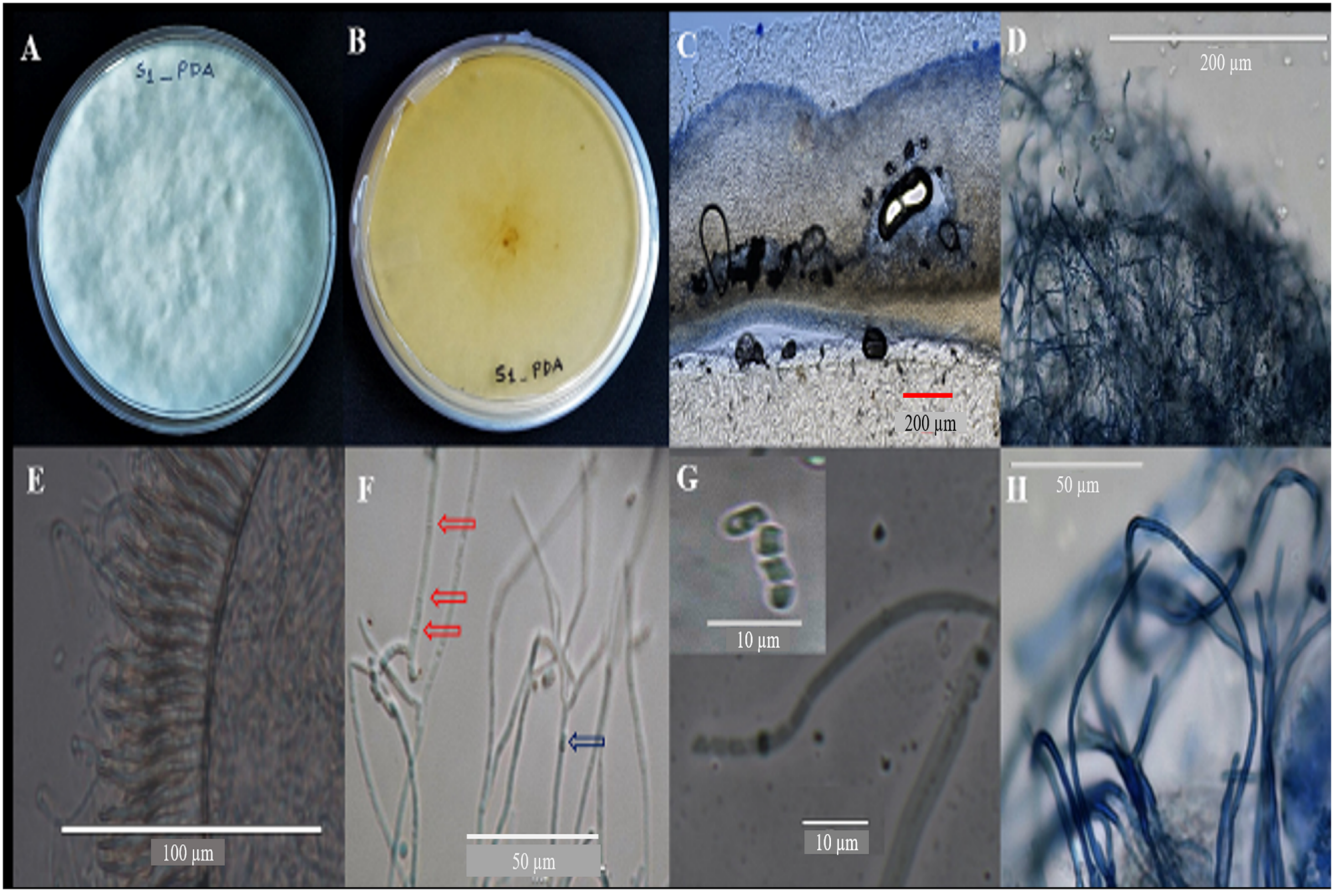
Macroscopic and microscopic characterization of the fungal isolate. 20 day old PDA plate showing growth of endofungus SDL-CO-2015-1, (A,B) surface and reverse of the colonies respectively (C) lateral section of texture, (D) cottony texture on PDA plate, (E) reticulate furrows in teeth pattern, (F) septum (red arrow) and dikaryotic hyphae (blue arrow), (G) holobasidia spore (upper-left corner), (H) low branching hyphae.

Surface color and texture of SDL-CO-2015-1 colonies were found to be white and cottony on a PDA plate (Fig 2A and 2D). These colonies were yellowish white on the reverse side (Fig 2B), widely effused, strongly adnate with the media surface, latitudinal spreading 650–750 μm thick (Fig 2C) with reticulate furrows; occurring in a teeth pattern, or in labyrinth form (Fig 2E), growing over the edge of the petri dish and becoming dark with age. No change in color was observed when dry. The intermediate layer of context was composed of densely gelatinized interwoven hyphae, opaque in nature. No zonate or radiate pattern was observed. Skeletal hyphae were found to be predominant, containing holobasidia (Fig 2G), basidiospores were 2.8 μm (Fig 2G upper-left corner) in size and cylindrical in shape. Septa were observed between the cells (Fig 2F, red arrow), filaments were found to be long, less branched with 2.5~3 μm thickness (Fig 2H). Basidia clavate was found to be tetraspored.

### Molecular identification and phylogenetic analysis of SDL-CO-2015-1

**Fig 3:**
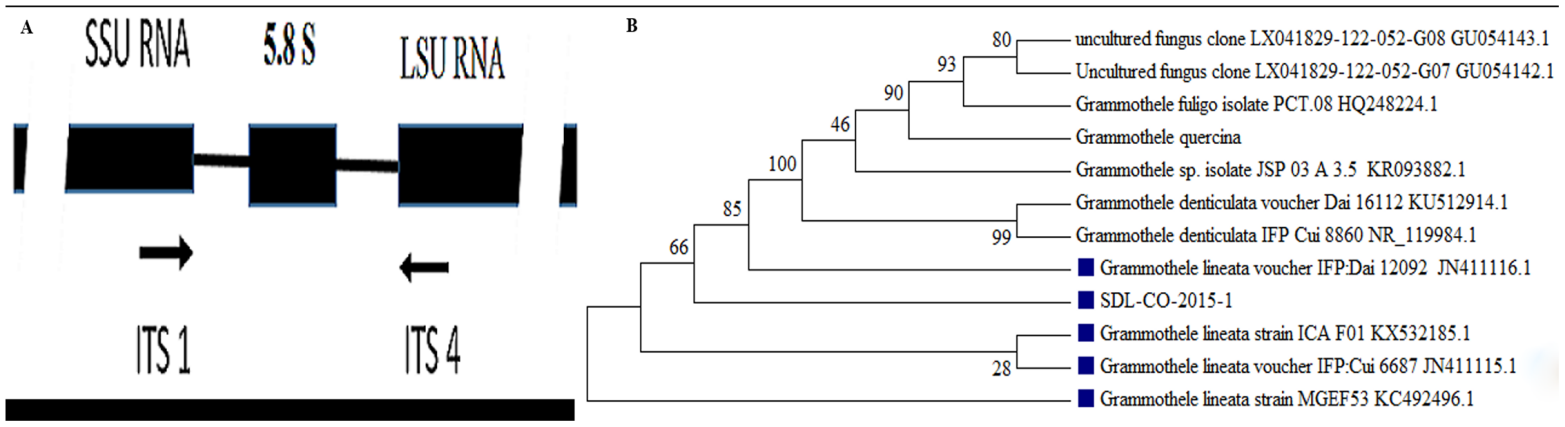
Phylogenetic tree. (A) 5.8S-coding sequence situated between the Small Sub-Unit-coding sequence (SSU) and the Large Sub-Unit-coding sequence (LSU) of the ribosomal operon. (B) Based on sequence homologies of the ITS region, SDL-CO-2015-1 was found to have identity with *Grammothele lineata*.

After isolation of fungal DNA, PCR with ITS (Fig 3A) specific primers gave a sharp and single band of approximately 650 bp (Fig 1B). The amplicon was then sequenced (Table A in S1 File). An online BLAST search of the sequence exhibited similarity with several species of the genus *Grammothele* and some uncultured endophytic fungi, where 99% identity was observed with *Grammothele lineata* (query coverage 97%). Phylogenetic relationship was determined through alignment and cladistic analysis of the homologous nucleotide sequences among the fungal species (Fig 3B). According to the evolutionary distance and morphological characters, SDL-CO-2015-1 was identified as *Grammothele lineata* belonging to the Polyporaceae family from the Basidiomycota phylum.

### Isolation and identification of taxol in extracts of *Grammothele lineata* SDL-CO-2015-1

**Fig 4:**
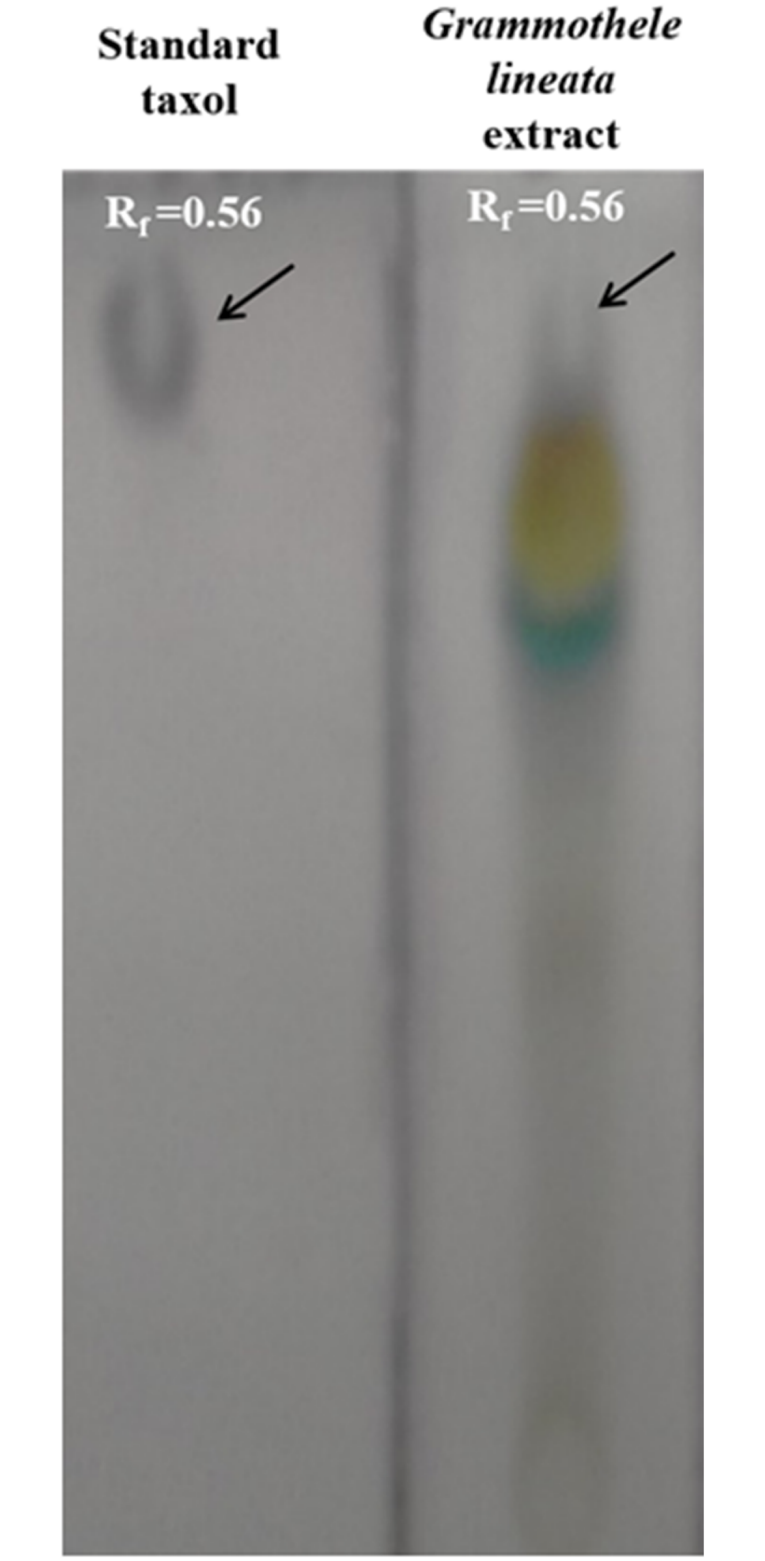
Thin layer chromatographic analysis of SDL-CO-2015-1 extract along with the standard taxol. TLC analysis of fungal taxol along with standard taxol in 1% vanillin/sulfuric acid (w/v). Standard taxol and fungal taxol show similar R_f_ value (0.56).

HPLC was used to isolate and purify taxol from a 21 day old culture of *Grammothele lineata* SDL-CO-2015-1. Presence of taxol in the fungal extract was first identified by TLC comparing with standard taxol. Extracellular extract of SDL-CO-2015-1 gave a band similar to standard taxol (R_f_ value both fungal and standard taxol was found to be 0.56) and displayed a dark grey color when sprayed with 1% vanillin/sulfuric acid (w/v) (Fig 4).

A characteristic peak with almost similar retention time as standard taxol was obtained for the extracellular extract of the fungus in HPLC, in a multistep gradient solvent system. The fungal taxol was next purified through semi preparative HPLC. It again exhibited the same retention time which was 31.3 min as the standard taxol (Figure A in S1 File).

**Fig 5:**
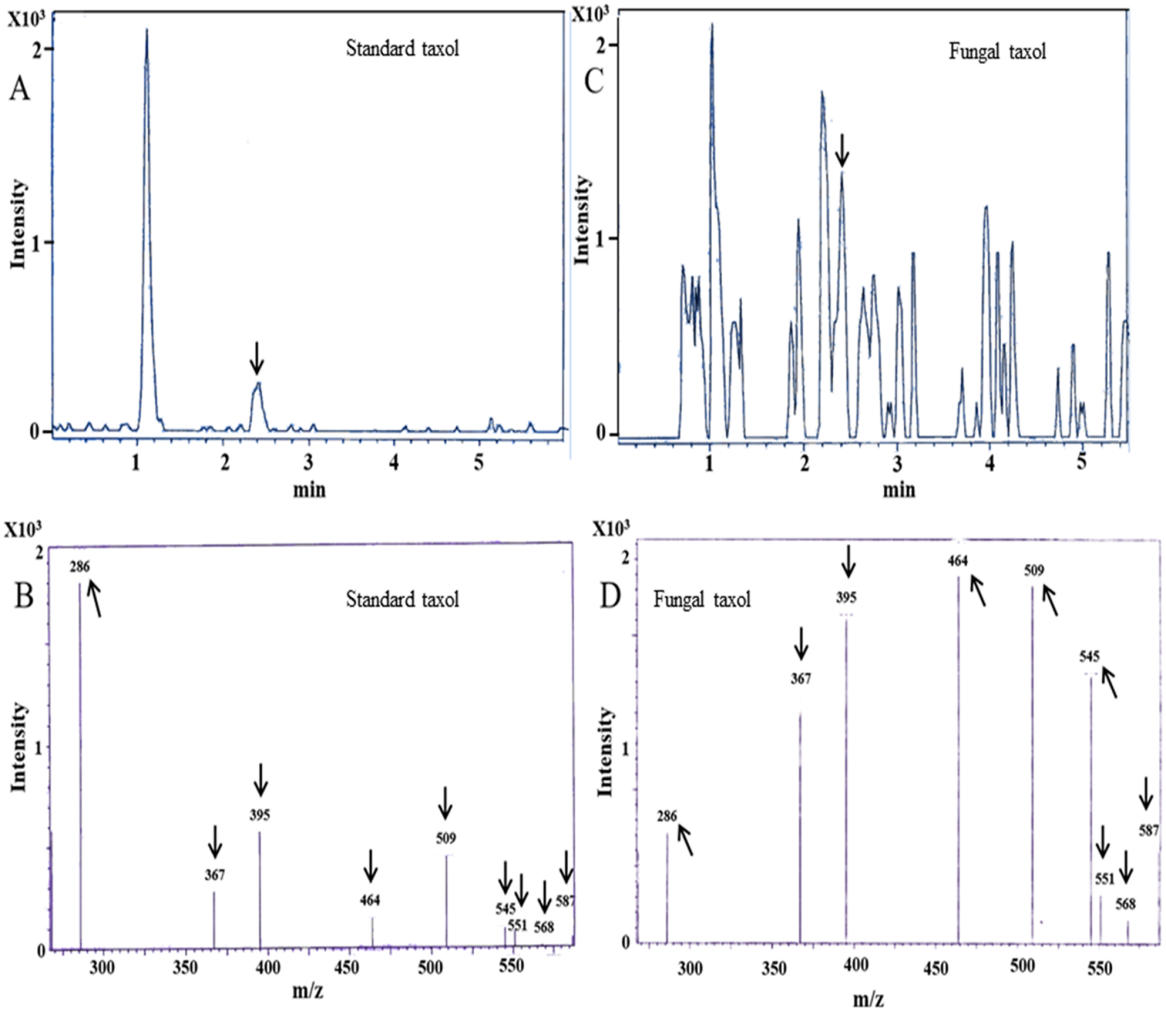
Analysis of taxol fragmentation pattern in LC-MRM-MS. (A) LC chromatogram for standard taxol. (B) MRM of standard taxol show characteristic peaks at m/z 286, 367, 395, 464, 509, 545, 551, 568 and 587. (C) LC chromatogram of fungal extracellular extract. (D) MRM of fungal taxol showed similar characteristic m/z with standard taxol at 286, 367, 395, 464, 509, 545, 551, 568 and 587.

LC-ESI-MS/MS scan of extracellular extract of SDL-CO-2015-1 gave a characteristic molecular ion peak (M+H)^+^ similar to taxol at m/z 854 (Figure B in S1 File). To confirm the presence of taxol, multiple reaction monitoring (MRM) was also performed using a triple quadrupole LC-MS/MS system, which monitors both the taxol ions and the characteristic fragment peaks of the precursor. We obtained daughter ion peaks at m/z 286, 367, 395, 464, 509, 545, 551, 568 and 587. These peaks were identical to standard taxol in multi reaction monitoring (MRM) (Fig 5). Both LC-ESI-MS/MS and LC-MRM-MS data appear to attest the presence of taxol in the fungal extract [29].

**Fig 6:**
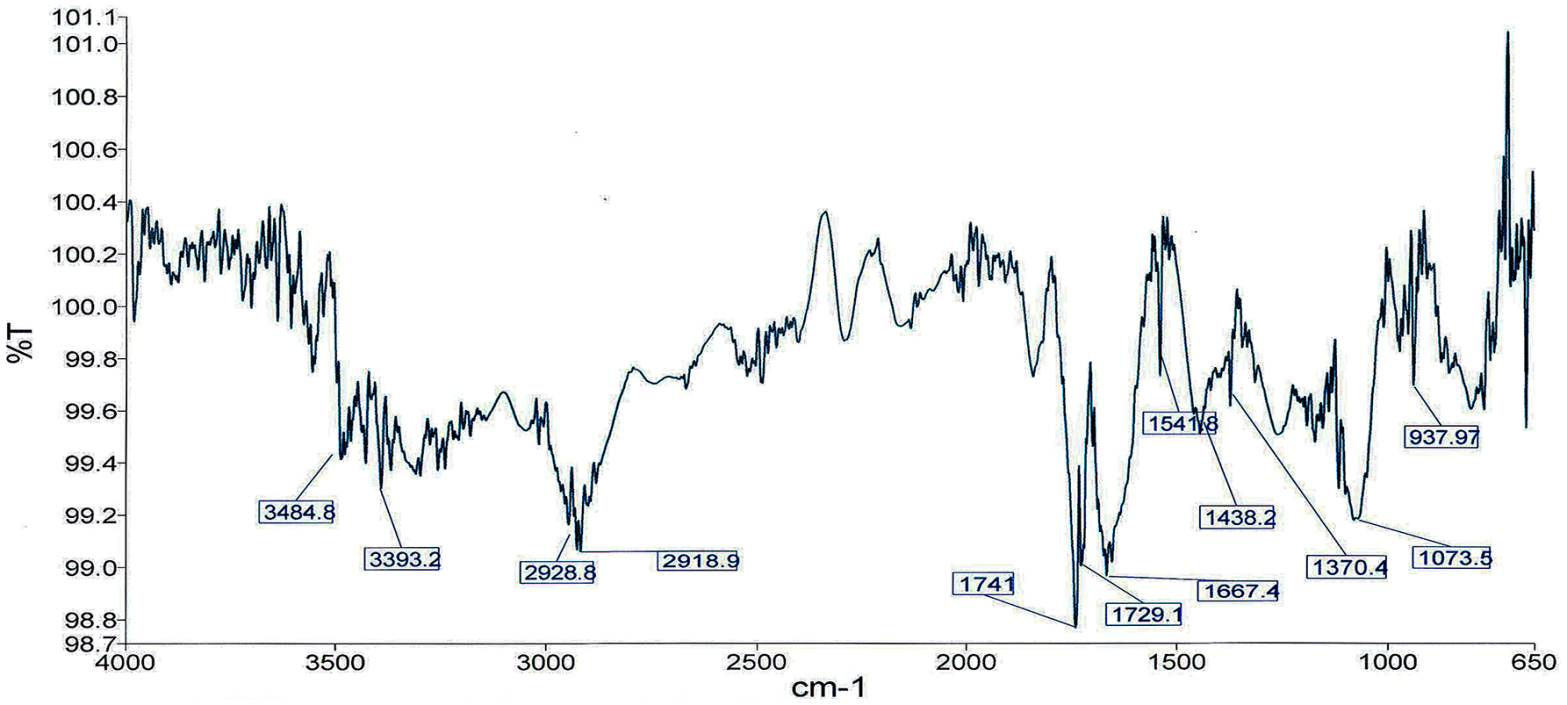
FTIR spectral analysis of fungal taxol.

The FTIR spectral data of fungal taxol from SDL-CO-2015-1 gave peaks at 3484.8 and 3393.2 cm^-1^ for hydroxyl (–OH) and amide (–C (O) NH–) group stretches respectively (Fig 6). Aliphatic CH stretch was observed at 2928.8 cm^-1^ and ester and ketone group (C = O) stretches were observed in the region of 1729.1 and 1741 cm^-1^ respectively. The aromatic ring (C = C) stretching frequency was observed in the region of 1667 cm^-1^. The—COO^-^ stretching frequency was observed at 1371.29. A peak observed at 1073 cm^-1^ was due to the presence of aromatic C, H bond [30].

In order to quantitate the amount of taxol produced by the endofungus, extraction from a 21 day old fungal culture in PDB media, was carried out using the protocol described in the methods section. The characteristic peak of fungal taxol at 31.367 min in HPLC was used to quantitate the amount of taxol produced by the fungus. Data obtained for the area of the peaks vs standard taxol concentrations were used to construct a standard curve (Figure C in S1 File) for estimating the amount of the amount of taxol produced by our endofungus. The yield of taxol from one liter of PDB medium was calculated to be 382.2 μgL^-1^

### Cytotoxic activity of purified fungal taxol

**Fig 7:**
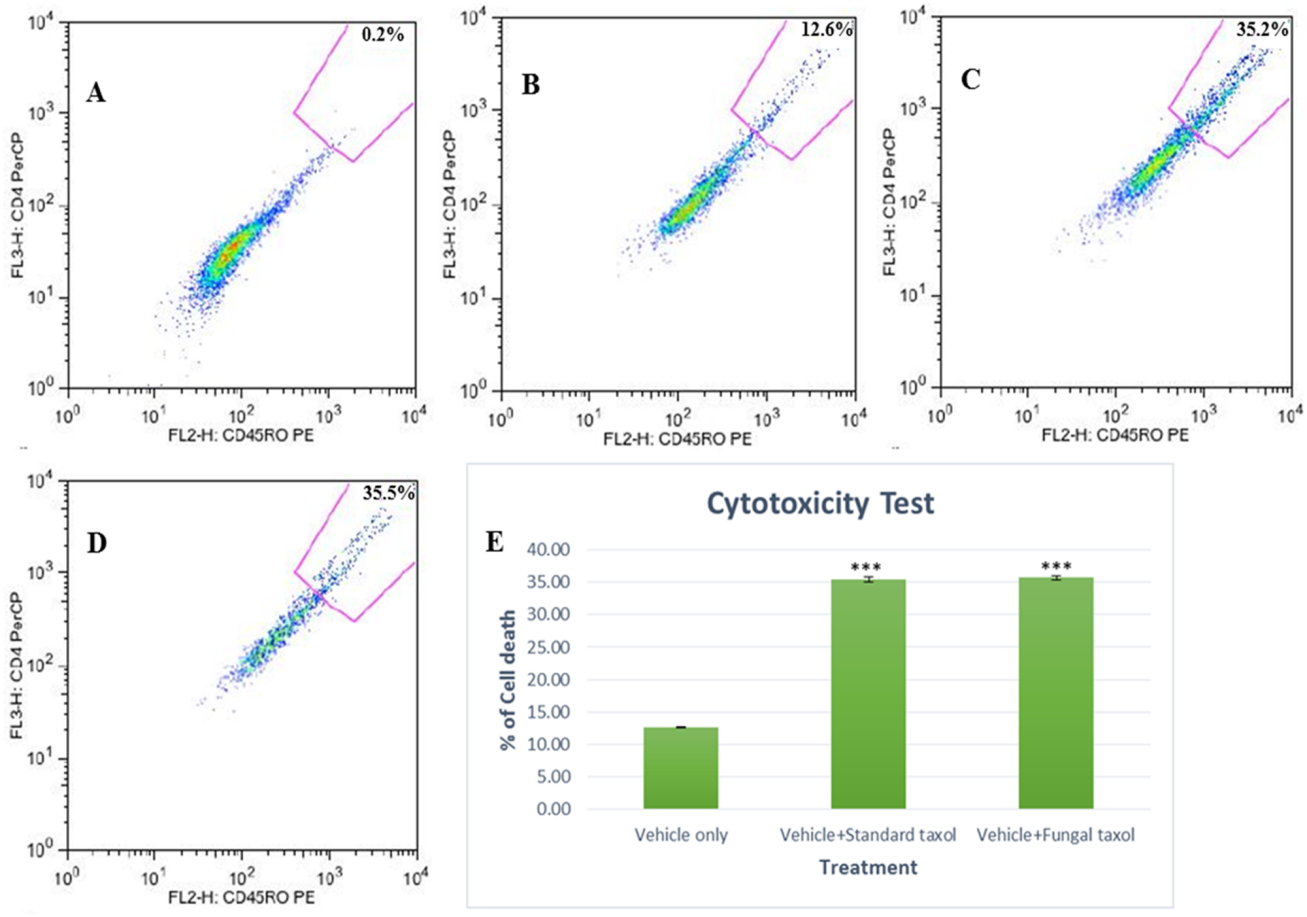
Induction of apoptosis in HeLa cell line treated with standard taxol, fungal taxol and 10% DMSO in DMEM media; as determined by PI staining through FACs analysis. (A) Untreated cells without PI staining (vehicle only). (B) Untreated cells with PI staining (vehicle only). (C) Cells treated with 0. 005μM of standard taxol. (D) Cells treated with 0. 005μM of HPLC purified fungal taxol. (E) Bar diagram shown as a FACs profile, standard taxol was used as a positive control and 10% DMSO in DMEM media as a vehicle control. The standard and fungal taxol gave almost same percentage of cell death which is significantly more than the vehicle control. These assays were performed for two independent events and replicated twice. (*** denotes p value<0.001, indicating high level of significance).

HPLC purified fungal taxol was tested for cytotoxic activity by apoptotic assay on HeLa cell line using PI staining. In FACs analysis 35% cell death was observed for both standard and fungal taxol (at a concentration of 0.005μM) whereas 12.64% cell death was observed for 10% DMSO in DMEM media (Fig 7).

### Antimicrobial activity of fungal extract

Antimicrobial activity was tested for an extract of *Grammothele lineata* SDL-CO-2015-1 against some pathogenic fungi and bacteria. Antifungal activity was assessed against two fungi, one plant pathogen, *Macrophomina phaseolina* and one opportunistic infectious pathogen, *Aspergillus fumigatus*. Only intracellular extract of SDL-CO-2015-1 gave a clear zone of inhibition against both the fungi (Figure D in S1 File) (Table 1). Antibacterial activity was assessed against a gram-positive (*S*. *aureus*) and a gram negative bacteria (*Burkholderia sp*). Clear zones of inhibition were found for both intracellular and extracellular extract against the both indicator strains (Figure D in S1 File) (Table 2). For each indicator strain the assays for two independent events were replicated twice.

## Discussion

Plant endophytic fungi are recognized as an important and novel resource of natural bioactive products. They offer a number of compounds with notable anti-tumorigenic applications [31] that are part of the war against cancer. Taxol, diterpene in nature, is among the most popular natural compounds that has been effectively used in cancer treatment as well as in neurodegenerative and polycystic kidney disorders [32]. So far, researchers have found more than 20 genera of endophytic fungi that produce taxol isolated from both *Taxus* and non-*Taxus* species and the wide range proves that both taxol-producing fungi and their hosts have considerable biological diversity [6]. With the help of genetic screening and chemical analyses we isolated an endophytic fungus, SDL-CO-2015-1 identified as *Gramothele lineata* harboring a taxol producing capacity from a jute plant, *Corchorus olitorius*. The isolated fungal taxol was also tested for the efficacy of its anticancer activity against HeLa cancer cell line.

A major significance of this study is the first ever identification of a fungus from the Basidiomycota phylum that is able to produce taxol. Till 2013, about 46 genera and 111 species of endophytic fungi producing antitumor components have been reported and taxonomically, nearly all of them belong to Ascomycota (96%) and only a paltry 3% have been found from the Basidiomycota phylum [33]. Interestingly almost all fungi reported to produce taxol are under the phylum Ascomycota, however, *G*. *lineata*, isolated as a jute endophyte belongs to Basidiomycota phylum and is the first ever taxol producing fungi to be reported from this phylum. Basidiomycetes are known to produce terpenoids as their primary class of secondary metabolites and often have unique structures not observed elsewhere in the natural world. On the other hand Ascomycota are mostly known for their polyketides and non-ribosomal peptides [34]. Several terpenoids from the Basidiomycota phylum show selective antitumor activity making them excellent candidates for cancer therapy [35]. Even though this phylum is considered the most sophisticated form of fungi, their diterpene biosynthetic pathways are largely unknown compared to Ascomycota and are predicted to be more diverse, complex and expected to yield more secondary compounds [36]. Basidiomycetes present a unique opportunity for the discovery of novel terpenoid biosynthetic routes leading to the development of compounds with new bioactivities [37]. Thus, the ability of SDL-CO-2015-1 to produce taxol does not come as a surprise and it is possible that the largely untapped fungal kingdom may reveal more members capable of producing diverse bioactive compounds-including antimicrobial compounds as found in this study. No previous work has reported the taxol producing ability of Basidiomycetes although a review has made such a claim [38] without providing any specific data to support the same.

Only one species under the genus *Grammothele*,—*G*. *fuligo* has been identified as an endophyte of oil palm [39]. Our isolate, SDL-CO-2015-1 was identified to be *G*. *lineata* based on ITS rDNA gene sequence and phylogenetic analysis. Phylogenetic analysis puts the isolate in the same clade with other *G*. *lineata* strains. Interestingly, this clade also includes some uncultured fungi which is not unexpected since a considerable number of fungi belonging to the Basidiomycota phylum frequently show poor growth under laboratory conditions [40]. According to the features described by Reck and Rosa [41], the basidiospores of *Grammothele lineata* are 2.5–3 μm thick matching with the basidiospores of our strain, SDL-CO-2015-1. However, our strain has a rare cylindrical shape rather than the more common ellipsoidal form. Macroscopically the surface texture and color of SDL-CO-2015-1 were found to be the same as that described for *G*. *lineata* by others [41]. Even though not much information is available on the morphology of *G*. *lineata* and the existing data are somewhat ambiguous, classical morphological identification together with molecular analysis suggests SDL-CO-2015-1 to be a new strain of *G*. *lineata*.

Biochemical techniques and spectrometric analyses used for the detection of taxol are tedious and time-intensive procedures. Therefore as a first choice it is convenient to screen for the presence of paclitaxel biosynthetic genes. A couple of reports have used such molecular approaches for the purpose of screening fungi [23, 42]. However, the biosynthetic pathway and the regulatory mechanism of taxol in fungi are still unknown. An intriguing question is whether the pathway is conserved among fungi and plants since at this stage, genes or pathways related to fungal taxol biosynthesis are still at large [43]. In this study genetic screening was carried out using three different genes that are vital to taxol biosynthesis (*ts*, *dbat* and *bapt*). Although *ts* is a rate limiting enzyme in the till known pathway both *dbat* and *bapt* are more diagnostic because more than ten enzymatic steps are required for the synthesis of taxol after *ts* [44]. Sequence information of these genes from fungi is scant, the reliance therefore is on taxol biosynthetic gene sequences of plant origin [45]. As a consequence primers used for screening taxol genes in endophytes are primarily based on available plant sequences. This appears to be justified if we take into consideration the recent genome analysis of the taxol-producing endophytic fungus *Penicillium aurantiogriseum* [46] which reveals sequence homology of key genes involved in taxol biosynthesis between plant and fungi. Origin of this pathway in these two physically associated groups appear to have been facilitated by horizontal gene transfer, HGT. However, another group has claimed to find no similarity in the pathways for taxol biosynthesis in fungi and plants when whole genome sequence and transcriptom analysis of two endophytic fungi from *T*. *brevifolia* were made. They envisage the biosynthetic pathway of taxol in endophytic fungi may have a distinctly different evolutionary pattern compared to plants [15]. Xiong, *et al*., 2013 have reported the ability of a specific fungus to produce taxol although the sequence they deposited for genes of the corresponding pathway gave negligible query coverage. Only the primer sequences were found to match [11]. However, the same fungus was found to produce taxol as confirmed by HPLC and LC-MS. This may explain why DNA sequence of individual genes of SDL-CO-2015-1 amplified with primers designed from conserved regions of corresponding taxol biosynthetic genes of plants yielded inconclusive results in *in silico* hybridization (blastn).

In this study, the presence of fungal taxol was screened primarily by TLC. Extracts of SDL-CO-2015-1 was found to possess an R_f_ value of 0.56, congruent with standard taxol and gave a spot dark grey in appearance when sprayed with 1% vanillin/ sulphuric acid. HPLC analysis of the extract gave a peak in reverse phase C18 column, with almost the same retention time as the standard taxol. Fragmentation pattern of fungal taxol in LC-ESI-MS/MS using LC-MRM-MS was found to be identical to the standard taxol [2]. Characteristic peak at m/z 545 and 587 are possibly for 10-deacetylbaccatin-III and baccatin-III respectively which are the main precursors of taxol, [29] attesting again the taxol production capability of the jute endofungus. In a simple nutrient media this fungus was found to produce approximately 382.2 μgL^-1^ of taxol, much higher than that reported for the endophytic fungus, *Taxomyces andreanae* in PDB [2]. This isolate capable of producing a considerable amount of taxol in a simple PDB media is expected to produce more if grown in M1D media supplemented with elicitors [47, 48].

When the isolated taxol was tested for its cytotoxicity, it was found to be relatively significant against the HeLa cancer cell line. *G*. *lineata* SDL-CO-2015-1 extract was also found to have both antibacterial and antifungal activity. It is not clear at this point in time if such bioactivities can be attributed to taxol. However, the antimicrobial activity of *G*. *lineata* is the first ever to be observed, and leads to the expectation that this Basidiomycete will actually become a new source of naturally effective bioactive compounds.

## Supporting information

S1 File Sequences of *G*. *lineata*, analytical data for fungal taxol identification, and bioassays of fungal extracts against indicator organisms. (DOCX)
