# Supplementary material for: An endophytic Basidiomycete, Grammothele lineata, isolated from Corchorus olitorius, produces paclitaxel that shows cytotoxicity
Source: PLoS One. 2017 Jun 21;12(6):e0178612. doi: 10.1371/journal.pone.0178612 (PMC5479517; doi:10.1371/journal.pone.0178612)
Supplement: S1 File — (DOCX) [file pone.0178612.s001.docx]

**Sequences of *G. lineata*, analytical data for fungal taxol identification, and bioassays of fungal extracts against indicator organisms**

**Table A:** *Grammothele lineata* sequence for ITS, ts and *dbat*

| Gene name | Sequence |
| --- | --- |
| ITS of *Grammothele lineata* | 5'-CTTCCGTAGGTGAACCTGCGGAAGGATCATTAACGAGTTTGAAACGGGTTGTAGCTGGCCTTCCGAGGCATGTGCACGCCCTGCTCATCCACTCTACACCTGTGCACTTACTGTGGGTTTCGATAGTCGTCGTGGGGTTCTACCTCGCGCGGCGATTCGGGGCTCACGTTTATTACAAACGCTTTAGTATCAGAATGTAAACCGCGATATAAACGCATTATATACAACTTTCAGCAACGGATCTCTTGGCTCTCGCATCGATGAAGAACGCAGCGAAATGCGATAAGTAATGTGAATTGCAGAATTCAGTGAATCATCGAATCTTTGAACGCACCTTGCGCTCCCCGGTATTCCGAGGAGCATGCCTGTTTGAGTGTCGTGAAATTCTCAACCTACAGACCCTTGCGGTTCTGCAGGCTTGGATTTGGAGGCTTGCTGGCTTTTACAGTCGGCTCCTCTTAAAAGCATTAGCTCGTTCCTTGCGGATCGGCTCTCGGTGTGATAATTGTCTACGCCGCGGCTGTGAAGCGTTTGGAGGGCTTCTAATCGTCTCGTTAGAGACAGCTTCATTGACATCTGACCTCAAATCAGGTAGGACTACCCGCTGAACTTAAGCATATCAATAAGCGGAGGAA-3' |
| *ts* | 5'-YNNTCAAACCCATGTCGAATTCAGAAGGCTTCCAGAAACAGGATCACCCGATCCGGGCTGAGCCAGGACCAGCCGGCCTAGCAGGGTTCAATGGGAACAAGGCGGTGGTTTGTTTTGTCAAGCCAACAGGGGAGGACGAATCAAAGACCCCAGGGACTCGAATTGGCAGCGCTTCTTCAGAGCTTGTCTGTCCATCGAGCAGGGTCCTCAATCGATGATAGGTCGGGTGACGCAGGAGGAGAGATCGCAAGGTGCTCAAATCTGCTCACTCAAGGTCCCCTTCCCCCGGGCCGTTGGCCGATCGTGGATCCCATGGAAAGGCGAAAGACCAATAATACGAGGACTAGATTCCAGAGTGTATGCAAACTTG-3' |
| *dbat* | 5'-AAAAGAAGAACCCTAAGATACATAGGANTCGNCAAACCTCAGAAGTGAATTGAGAAGGAGGAGACGACAANCGAAACGAGCAATACTTCACGTATTTAGGTGCCTTGTAGGCTTGTAGGTACTTTGAGTCTGGGGTAATGGACATCCAACATGAACGTAAAGCCACAATGAATTGGACAAGCACCAAAGCTCATTGTTAGGCCACCGGAAGCAATGTTTCAGGTTTGCCCCATGTTCCATAGCCACCTTGGAGAGCTCCGAACAAGGGAGCGAAGCACATGTAACAATTGACCATGGCTGGGCAACCGATGACACCTGCCTAACCAAGGGGCGCCAGCACCACCCGGTATTATAGTGCCTGAGACCACACGTGTAGGATCCACACACATGTGATGGTCTAGGAGCAGGCCATGTGATGGACTAGGAGCAGGCCATGTGATGGACTAGGAGCAGGCCCTGTGATGGACTAGGACCAGGCCCTGTGATGGACTAGGAGCAGGCCATGTGAAGGACTAGGAGCAGGCCAAATCATGGNCAATCANCCCGNCANTCTTGGATTAGGAACTGT-3' |

**I**


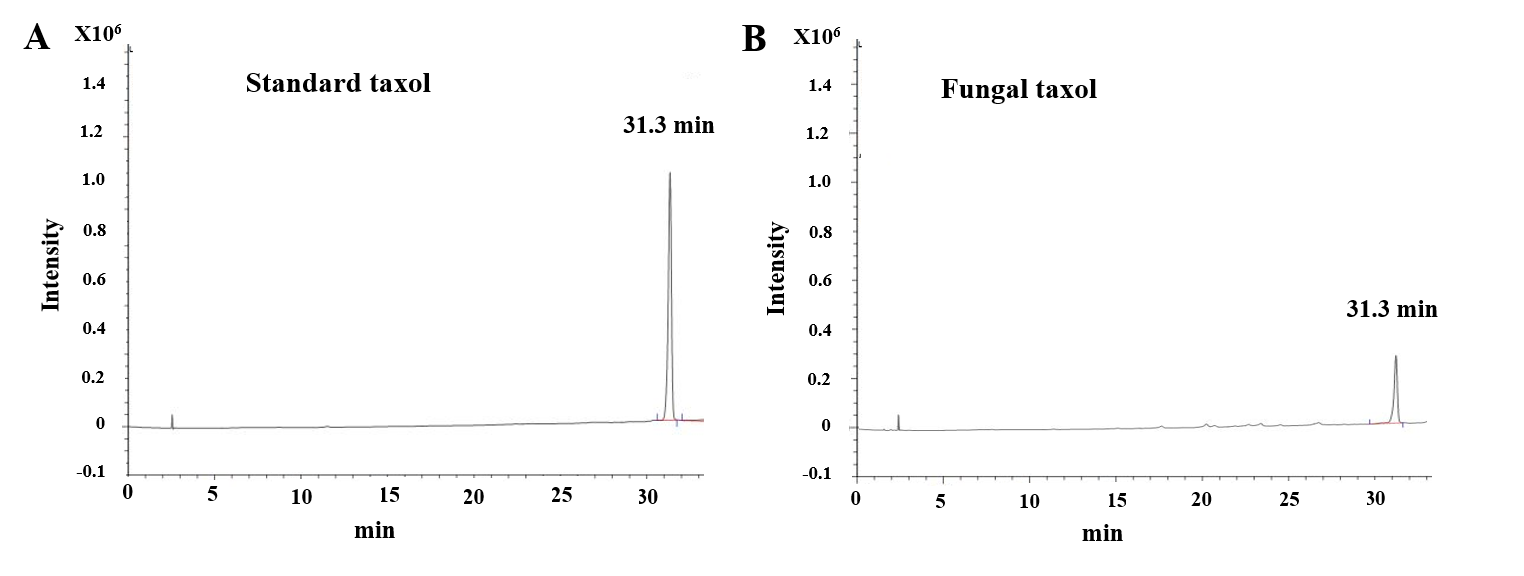


**II**

**Fig A.** High performance Liquid Chromatographic analysis of fungal taxol and standard taxol in 227 nm (Rt = 31.3 min)

(I) Standard taxol showed a characteristic peak at 31.3 min. (II) HPLC purified fungal taxol showed a characteristic peak at 31.3 min.


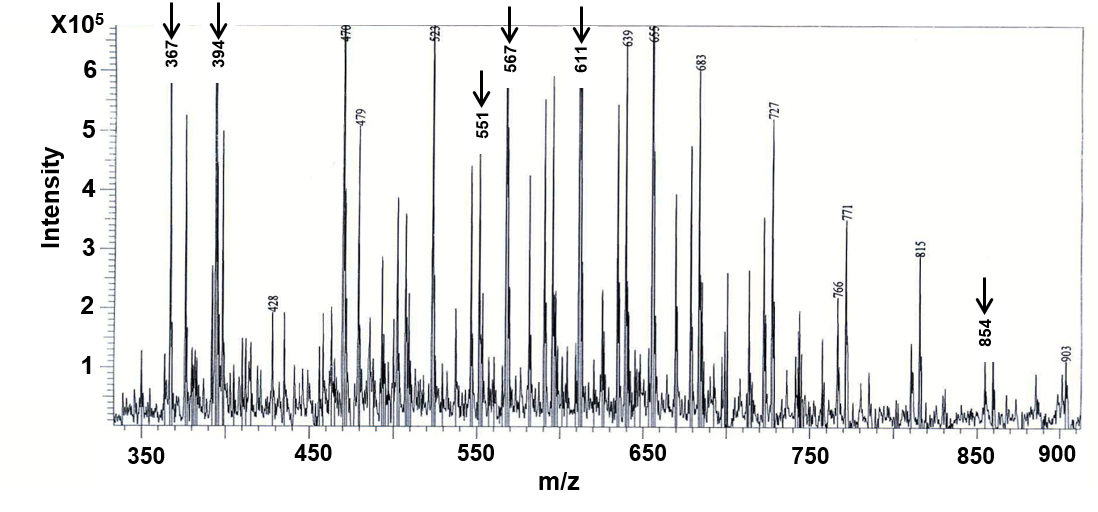


**Fig B**. LC/ESI mass spectrometric (scan) analysis of SDL-CO-2015-1 extract.

Characteristic peak of SDL-CO-2015-1 extract similar to standard taxol at m/z 854.


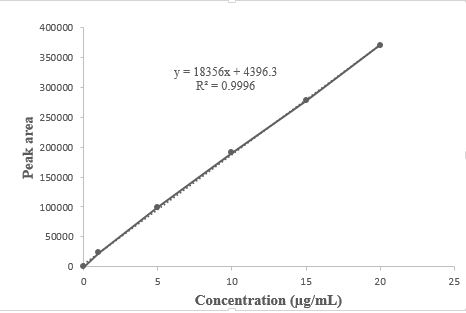


**Fig C.** Calibration curve of standard taxol for fungal taxol quantitation.

Concentration of taxol was found to be directly proportional to area of the peak at 227 nm.


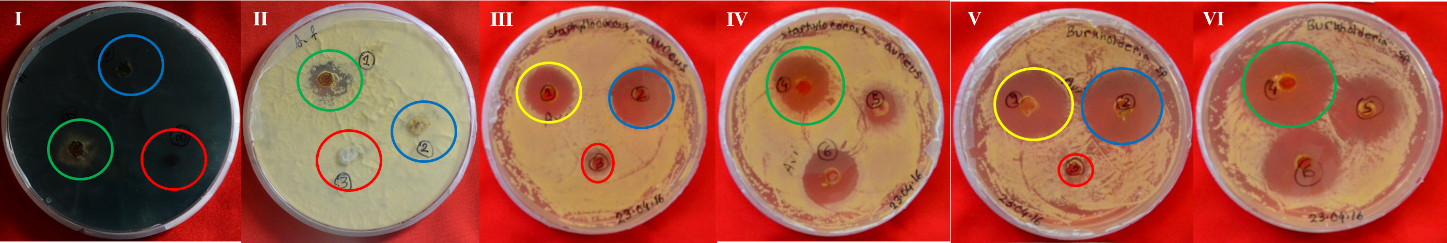


**Fig D**. Antimicrobial screening of SDL-CO-2015-1 extract.

(I) *Macrophomina phaseolin*a, (II) *Aspergillus fumigatus*, (III, IV) *S. aureus* and (V,VI) *Burkhoderia sp.*. [Green circle: intracellular extract, blue circle: extracellular extract, red circle: methanol, yellow circle: ampicillin].
